# Differences in Physiological and Agronomic Traits and Evaluation of Adaptation of Seven Maize Varieties

**DOI:** 10.3390/biology13120977

**Published:** 2024-11-26

**Authors:** Shuqi Ding, Dan Zhang, Ying Hao, Mengting Hu, Huijuan Tian, Kaizhi Yang, Guolong Zhao, Ruohang Xu, Wentao Du

**Affiliations:** 1 College of Agriculture, Tarim University, Alar 843300, China; 1011219106@stumail.taru.edu.cn (S.D.); hy18386110782@163.com (Y.H.); humtww@163.com (M.H.); wmzthj@163.com (H.T.); yangkaizhi2022@163.com (K.Y.); glzhao0311@126.com (G.Z.); 17861086520@163.com (R.X.); t2273266657@163.com (W.D.); 2 Key Laboratory of Genetic Improvement and Efficient Production for Specialty Crops in Arid Southern Xinjiang of Xinjiang Corps, College of Agronomy, Tarim University, Alar 843300, China

**Keywords:** maize, variety, selection, comprehensive evaluation

## Abstract

To better understand the growth adaptability of various maize varieties to the climate of the Alar region in Southern Xinjiang Province, an experiment was conducted using seven distinct maize varieties as test materials. A one-way randomized block design was applied to both experimental groups. In 2021 and 2022, a total of 19 indicators were observed for comparative analysis, including antioxidant enzyme activities and agronomic traits. Principal component analysis and cluster analysis were used to evaluate the adaptability of the maize varieties. The findings revealed that: (1) All seven maize varieties exhibited robust growth, with notable differences in their respective trait profiles. Specifically, the yield traits of Jin’ai 588 and Denghai 3672 showed relatively consistent performance over the two-year period. (2) Five principal components (100-kernel weight, bald tip length, catalase (CAT), number of leaves, and angle of leaf pinch at the ear) were extracted from the 19 traits via principal component analysis, with a cumulative contribution rate of 84.689%. This represented the majority of the information regarding the seven maize varieties. After calculating the comprehensive index F value, the results indicated that Xinyu 66 and Denghai 3672 had high composite scores, suggesting high production potential and suitability for cultivation in this region. Conversely, Xinyu 24 showed the lowest composite score, indicating that it is not suitable for planting in this area. (3) Ultimately, the seven maize varieties were categorized into three groups through cluster analysis; this is the same as the result of principal component analysis. This classification provides a reference for the promotion and utilization of different varieties in the southern border region and aims to optimize the comprehensive trait selection of the varieties studied.

## Simple Summary

In order to understand and utilize the adaptability of different maize varieties in the Alar region of Southern Xinjiang, 19 agronomic and physiological traits were measured using seven maize varieties as experimental materials. By using variance analysis, principal component analysis, and cluster analysis, the adaptability of maize varieties was evaluated comprehensively, and five traits and maize varieties suitable for planting in Southern Xinjiang were selected, which provided a reference for the promotion and utilization of different maize varieties in the Alar region of Southern Xinjiang.

## 1. Introduction

Maize (*Zea mays* L.) is an important cash and food crop, as well as a key energy source, cultivated to meet human needs [1]. Xinjiang is an agricultural irrigation area with abundant light and heat resources. Maize plays a vital role in the grain crop sector, ranking as a primary crop after wheat. The producing areas are concentrated in the oasis plain in the north and southwest of the Tarim Basin, accounting for 43% of the total corn production in Xinjiang [2]. As China’s population continues to grow and animal husbandry experiences rapid expansion, the demand for maize is rising. In 2021, the area planted with maize in Xinjiang expanded by 4.67 × 10^4^/hm^2^, accounting for over 40% of the national share [3]. However, due to the great differences in ecological conditions in different regions of Xinjiang, different ecological conditions put forward different requirements for corn varieties [4]. Consequently, it is essential to closely examine the growth adaptability of various maize varieties in the Aral region of Xinjiang.

Maize exhibits significant variation in biological characteristics across different regions, including morphological traits, physiological traits, and yield-related traits. The crop is sensitive to a range of environmental factors, such as temperature and the level of mechanization employed in cultivation [5]. As a result, maize varieties need to have excellent adaptability and stable performance. Studying the growth characteristics of maize varieties is a key method for identifying superior germplasm [6]. The principal agronomic traits of maize include plant height, ear height, leaf area, number of leaves, and thousand-kernel weight [7,8]. A correlation has been identified between yield and agronomic trait indicators. Yield is significantly and positively correlated with the leaf area index (LAI), plant height, and stem thickness, indicating that an increase in LAI, plant height, and stem thickness could lead to an increase in yield [9]. The physiological characteristics of maize are also important for its growth and development. Previous research has shown that maize is a stress-sensitive crop. When excessive stress is high, maize growth is inhibited, metabolic activity is weakened, and severe yield reduction and crop death can occur [10]. Therefore, improving the ability to resist coercion of maize varieties is a key objective. It has been shown that the elevated activity of superoxide dismutase (SOD) and peroxidase (POD) in maize can effectively scavenge reactive oxygen species and prevent membrane lipid peroxidation, which plays a beneficial role in maize’s defense against unfavorable environmental conditions [11]. Under adverse conditions, the activity of POD and SOD increases, and the concentration of malondialdehyde (MDA) rises. The level of intracellular MDA varies between different maize varieties [12]. Thus, determining the indicators of maize leaf oxidase activity is important for selecting superior varieties in maize breeding.

To accelerate the application of local maize germplasm resources in production, genetic diversity analysis, comprehensive evaluation of traits, and screening for target traits are essential. This approach can address the issues of the limited availability of high-quality germplasm and the low efficiency of resource utilization in maize breeding in China [13]. The Genotype Main Effect plus Genotype-by-Environment Interaction (GGE) biplot is a tool used to study genotype and genotype-by-environment interaction (G × E) in different environments by focusing on environmental yield stability [14]. GGE biplots use linear models to account for G × E effects, visualize the stability of genotypes and their optimal expression in specific environments, and more intuitively and quantitatively analyze yield differences among varieties. This method has been widely applied to crops such as rice [15,16], wheat [17], maize [18,19], and cotton [20,21]. Li et al. [22] utilized 487 maize germplasm resources as research materials, ultimately screening 15 with superior ear traits. Zheng et al. [23], Yao et al. [24], and Cai et al. [25] analyzed the genetic diversity and population structure of local varieties in Southwest China. Their findings showed that this region holds the largest variety of local germplasm resources and the highest level of genetic diversity [26]. Cluster analysis is also commonly used to classify maize diversity, and the classification of related phenotypic traits provides a reference for utilizing germplasm resources [27,28,29].

Previous studies have conducted adaptive analyses of maize yield. This experiment builds upon the work conducted in 2021~2022, which involved the determination and comparative analysis of the agronomic and physiological traits of seven maize varieties from 2021 to 2022 to explore the interaction effect between variety and year, as well as the stability between different years. The traits were evaluated comprehensively by principal component analysis and cluster analysis. The aim is to identify the varieties suitable for cultivation in the Alar region of South Xinjiang and similar climates.

## 2. Materials and Methods

### 2.1. Experimental Materials

The maize accessions utilized in this study were from 5 provinces, as shown in Table 1.

### 2.2. Experimental Design

Field trials were conducted in 2021 and 2022 at the Agronomy test base in the East District of Tarim University using a completely randomized experimental design with three replications. Seeds were sown in moist soil on 20 April 2021 and 21 April 2022. The experimental area covered 10.8 m^2^, with a plant spacing of 0.3 m, row spacing of 0.5 m, and three rows planted for each material.

### 2.3. Research Area Climate Characteristics

South Xinjiang is located in the southern region of the Xinjiang Tianshan Mountain range, characterized by a typical continental arid climate with annual precipitation ranging from 20 to 100 mm. The average temperature in the South Xinjiang Plain varies between 10 °C and 13 °C, enjoying a frost-free period of 200 to 220 days. The area experiences significant diurnal temperature variations and low rainfall due to its arid conditions. Alar City, situated in the southern part of the Xinjiang Uygur Autonomous Region of China, lies on the northern edge of the Taklamakan Desert and the upper reaches of the Tarim River (80°30′~81°58′ E; 40°22′~40°57′ N). The city’s climate is classified as warm-temperate extreme continental arid desert. The average annual effective accumulated temperature at or above 10 °C is 4541.4 °C, with average annual sunshine hours ranging from 2556.3 to 2991.8 h [30]. Alar City benefits from ample light and heat, although it is occasionally subjected to dust storms.

### 2.4. Irrigation and Fertilizer Application in the Trial Area

The soil texture at the test site was sandy loam texture, and the basal fertility of the 0–20 cm soil layer was assessed. The soil organic matter content was 7.64 g/kg, while the alkaline-dissolved nitrogen, available phosphorus, and available potassium contents were 26.6 mg/kg, 18.7 mg/kg, and 141 mg/kg, respectively. Irrigation was provided using a tube with two rows of under-membrane drip tubes, aligned with the water requirements of maize. The preceding crop at the site was cotton. Prior to sowing, 525 kg/ha of compound fertilizer was applied, followed by 270 kg/ha of urea one month after seedling emergence. Harvest took place in early October of the same year, once the crops reached maturity. Fertilizer application, cultivation management, and pest control practices in the experimental field mirrored those used under standard field conditions.

### 2.5. Comparison of April–October Climate in the Test Area for Two Years

**Figure 1:**
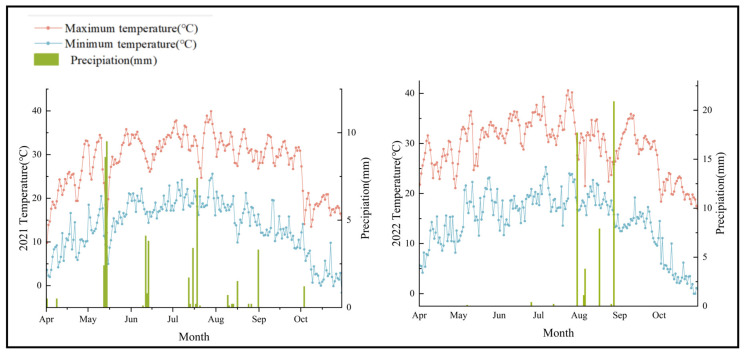
Comparison of temperature and precipitation for April–October 2021–2022.

Peak temperatures in both 2021 and 2022 occurred between July and August. In 2021, rainfall was primarily concentrated in May and July, whereas in 2022, it was mainly concentrated in August and September. The distribution of precipitation across the two years showed a significant difference (Figure 1)

### 2.6. Experimental Methods and Measurement Indicators

In this study, the phenotypic characteristics of 7 maize accessions were assessed in field conditions as per the Specification and Data Standard for Maize Accessions Description [31].

The following morphological traits were assessed at maturity for the seven maize varieties: plant height (cm) [32], ear height (mm) [33], stem diameter (mm) [34], ear leaf length (cm) [34], ear leaf width (cm) [34], leaf number (piece) [35], and stem–leaf angle (°) [36].

Yield components: A total of 30 ears from each maize variety were harvested to determine indicators such as ear length, ear diameter, cob diameter, ear tip-barrenness, row number per ear, kernel number per row, 100-kernel weight, and ear weight [37,38].

Physiological Indices: The assays for MDA [39], SOD [40], POD, and PRO were conducted using assay kits from Suzhou Keming Biotechnology Co., Ltd., Suzhou, China. Fresh samples weighing 0.1 g were homogenized in 1 mL of extraction solution in an ice bath. The samples were then shaken for 10 min in a water bath at 95 °C and centrifuged at 25 °C for 10 min, with the supernatant collected. After cooling, absorbance was measured using a spectrophotometer to calculate the PRO content. For POD activity measurement, 0.1 g of fresh sample was homogenized in an ice bath, centrifuged at 8000× *g* at 4 °C for 10 min, and the supernatant was placed on ice for analysis. POD activity was calculated using a spectrophotometer.

### 2.7. Data Analysis and Methods

The two-year data were analyzed by using the average value of the plot. After testing for the homogeneity of variance (Bartlett test), a multi-year joint ANOVA was performed using the PROC GLM program of the SAS 9.4 M3. The joint ANOVA fixed effect model is as follows [41]:

Yver = μ + Ee + Er(e) + Gv + EGev + εver

Yver is the observed value of the v variety in r repetitions in the e year; μ is the overall mean value of the test. Ee is the year effect; Er(e) is the year block effect; Gv is the variety effect; EGev is the interaction effect between year and variety. εver is the error term.

Descriptive statistics including the maximum, minimum, mean, standard deviation, and coefficient of variation of 19 indicators of the seven maize materials were performed using the Microsoft Excel 2021 software [42,43].

A one-way analysis of variance (ANOVA) was performed on the 19 indicators using the GraphPad Prism version 9 software and bar graphs were plotted [44].

Double standard analyses of the morphological traits, physiological traits, and yield-related traits of the seven maize varieties over two years were performed using the GenStat 9.2 software [45].

Correlation analyses of yield-related traits were carried out using R.4.33 [46].

Principal component analysis was carried out using IBM SPSS Statistics 27.0 on the mean data of the 19 traits over two years using the Varimax method [46], the data were standardized using the affiliation function, and the formulae were applied to calculate the composite rating values [47].

Systematic cluster analysis, squared Euclidean distance, and the sum of squares of deviations method were performed using the OriginPro2024 software [48].

## 3. Results

### 3.1. Mean Square and Significance Analysis of Combined Variance Analysis of Different Traits of Maize Varieties

The results of the joint ANOVA (Table 2) demonstrated that with the exception of the number of leavers, 18 traits exhibited highly significant differences between the years and varieties. This indicates that there were considerable variations in environmental conditions between the two years. With the exception of the kernel number per row, the variety and year interactions reached significant levels for the remaining 18 traits, indicating the prevalence of varietal and year interactions.

### 3.2. Diversity Analysis of Morphological Traits of Maize Varieties

**Figure 2:**
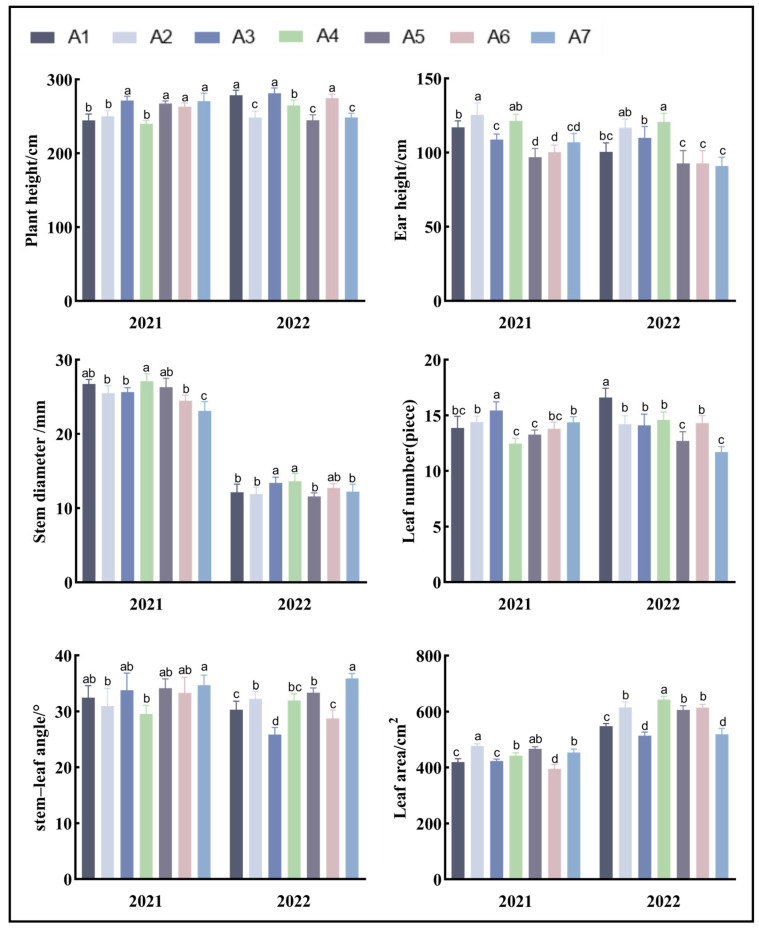
Analysis of the morphological traits of the maize plants. Note: In the figure, (a–d) markers of significance of differences at the 0.05 level. A1, A2, A3, A4, A5, A6, and A7 represent Jin Ai 588, Zheng Dan 958, Xin Yun 66, Xin Yu 24, Deng Hai 3672, Gan Xin 2818, and Xin Yu 81, respectively.

According to the diversity analysis of the morphological traits of the maize varieties (Table 3), the morphological traits of the different maize varieties were significantly different. The analysis of the coefficients of variation for each index revealed that except for the ear height index, the coefficients of variation for the plant traits in the different maize varieties were below 10%, and the ear height index was more strongly suggesting substantial differences between the varieties. The stem diameter of the corn varieties in Figure 2 was significantly larger in 2021 than in 2022.

### 3.3. Diversity Analysis of Physiological Traits of Maize Varieties

**Figure 3:**
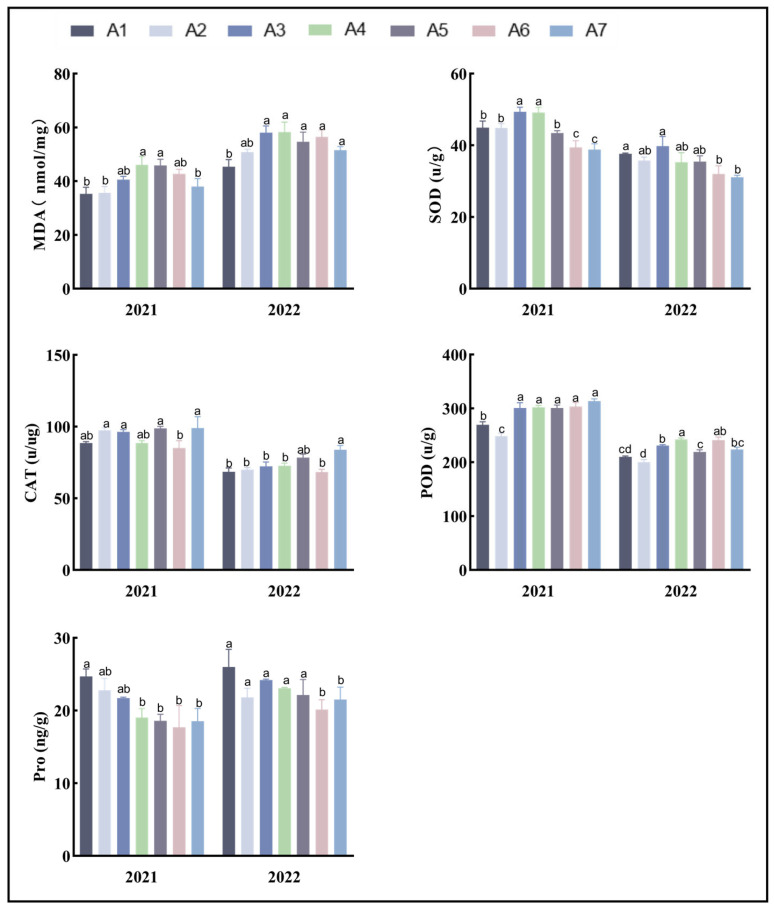
Analysis of the physiological traits of the maize plants. Note: In the figure, (a–d) markers of significance of differences at the 0.05 level. A1, A2, A3, A4, A5, A6, and A7 represent Jin Ai 588, Zheng Dan 958, Xin Yun 66, Xin Yu 24, Deng Hai 3672, Gan Xin 2818, and Xin Yu 81, respectively.

According to the diversity analysis of the maize varieties (Table 4), the activities of SOD and POD in A3 were relatively high. The CAT and POD activities of A5 and A7 were relatively high. The coefficient of variation of each index was less than 10%. A1 has the lowest MDA content, but the Pro content is higher in these two years (Figure 3), which is different from the other varieties.

### 3.4. Comparative Analysis of Yield-Related Traits in Different Maize Varieties

**Figure 4:**
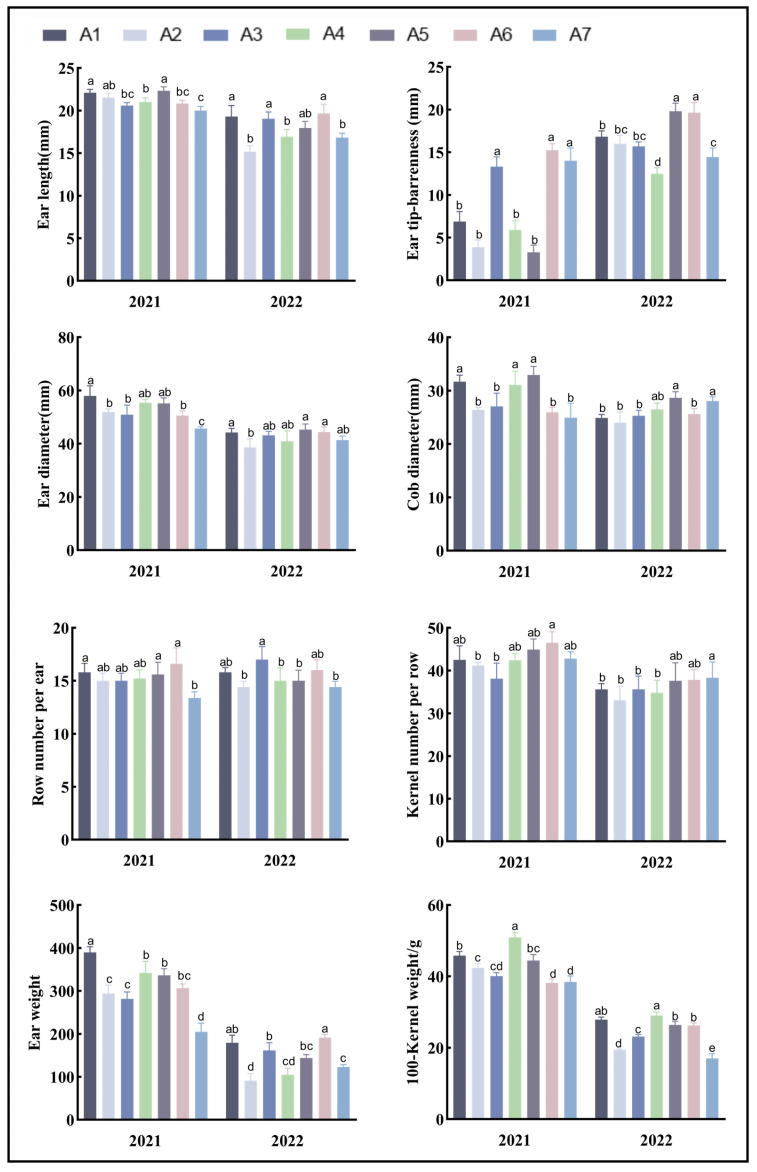
Analysis of the yield-related traits of the maize plants. Note: In the figure, (a–e) markers of significance of differences at 0.05 level. A1, A2, A3, A4, A5, A6, and A7 represent Jin Ai 588, Zheng Dan 958, Xin Yun 66, Xin Yu 24, Deng Hai 3672, Gan Xin 2818, and Xin Yu 81, respectively.

When comparing the yield-related traits of the different maize varieties (Figure 4), A1 showed superior performance in ear length and ear weight. A5 excelled in cob diameter and kernel number per row. An analysis of the coefficients of variation (Table 5) for the yield-related traits of the seven maize varieties revealed that aside from the ear tip-barrenness, ear weight, and 100-kernel weight, the coefficients of variation for most yield-related traits were below 10%. This indicates that these three traits are greatly affected by the variety. As can be seen from Figure 4, the ear weight and 100-kernel weight of the different maize varieties in 2022 were significantly lower than those in 2021. This could be due to a relatively dry environment in 2022.

### 3.5. Stability Analysis of Agronomic Traits and Physiological Traits of Maize Varieties

#### 3.5.1. A Stability Analysis of Morphological Traits in Different Maize Varieties

**Figure 5:**
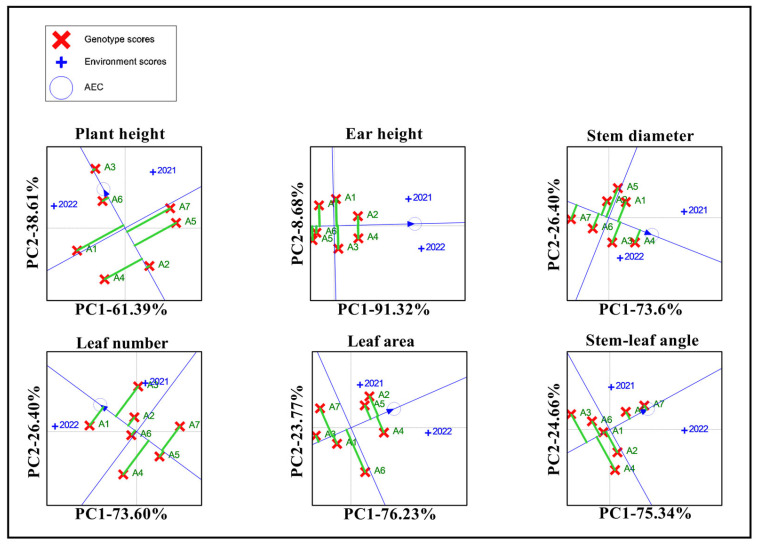
GGE biplot of the morphological traits of the different maize varieties. Note: A1, A2, A3, A4, A5, A6, and A7 represent Jin Ai 588, Zheng Dan 958, Xin Yun 66, Xin Yu 24, Deng Hai 3672, Gan Xin 2818, and Xin Yu 81; +2021 represents the planting environment in 2021; and +2022 represents the planting environment in 2021, respectively.

The stability of the morphological traits for the seven maize varieties was evaluated using GGE biplots. The varieties positioned to the right of the mean environmental vertical axis showed better performance than the overall average, while those to the left of the vertical axis performed below the average. The dashed segments perpendicular to the mean environmental axis represent the stability of the varieties, with shorter distances indicating greater stability in morphological traits and longer distances reflecting increased instability. The GGE biplots (Figure 5) revealed that plant height and stem diameter were above the overall environmental average for all four varieties, with A3 and A6 displaying greater stability. Additionally, four varieties, A5 and A6, exhibited ear heights above the environmental average. The leaf number was higher than the average in three other varieties, with A5 demonstrating greater stability. Leaf area was higher than the environmental average in three varieties, with A2, A5, and A6 exhibiting greater stability. A3, A5, and A6 exhibited a relatively high level of stability across all the traits. The dashed lines of A3 and A5 perpendicular to the environmental axis are relatively short, and the morphological characteristics are relatively stable in these two years.

#### 3.5.2. A Stability Analysis of Physiological Traits in Different Maize Varieties

**Figure 6:**
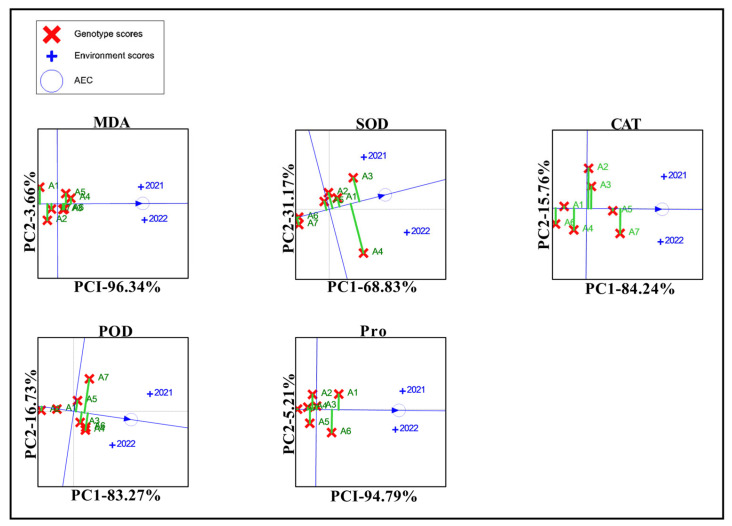
GGE biplot of the physiological traits of the different maize varieties. Note: A1, A2, A3, A4, A5, A6, and A7 represent Jin Ai 588, Zheng Dan 958, Xin Yun 66, Xin Yu 24, Deng Hai 3672, Gan Xin 2818, and Xin Yu 81; +2021 represents the planting environment in 2021; and +2022 represents the planting environment in 2021, respectively.

The stability of the physiological traits for the seven maize varieties was evaluated using GGE biplots (Figure 6). In contrast, the remaining six varieties, with the exception of A1, exhibited greater stability. SOD stability was superior in five varieties, with the exception of A3 and A4. CAT content was above the environmental average in all four varieties, with A1 and A5 exhibiting the greatest stability. The number of leaves was less than the overall environmental average in A1 and A2, with A3 and A5 demonstrating greater stability. The five physiological traits of A3 and A5 were all higher than the average value of the overall environment, and the dashed lines perpendicular to the environmental axis were shorter, so the physiological traits were stable.

#### 3.5.3. A Stability Analysis of Yield-Related Traits in Different Maize Varieties

**Figure 7:**
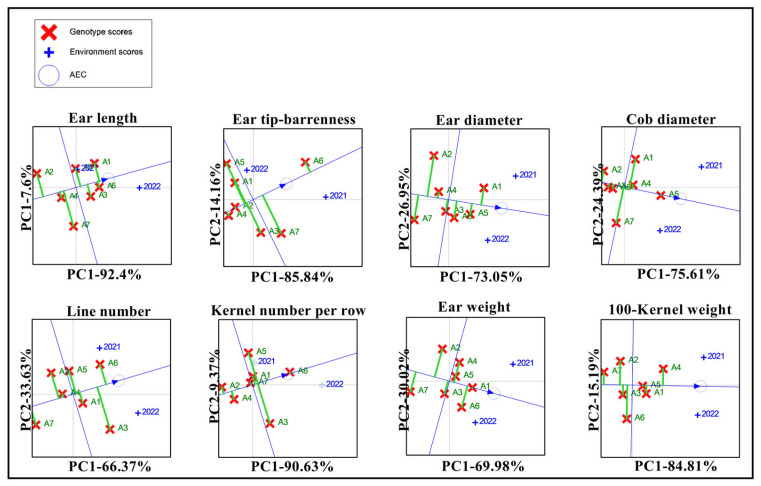
GGE biplot of the yield-related traits of the different maize varieties. Note: A1, A2, A3, A4, A5, A6, and A7 represent Jin Ai 588, Zheng Dan 958, Xin Yun 66, Xin Yu 24, Deng Hai 3672, Gan Xin 2818, and Xin Yu 81; +2021 represents the planting environment in 2021; and +2022 represents the planting environment in 2021, respectively.

The stability of the yield-related traits for the seven maize varieties was evaluated using GGE biplots. The GGE biplots (Figure 7) show that four varieties exhibited ear length, row number per ear, and ear weight exceeding the overall environmental average, with A1 and A5 being the more stable varieties. Additionally, two varieties, A6 and A7, displayed ear tip-barrenness above the environmental average. Four varieties had cob diameters greater than the overall environmental average, with A3 and A5 showing the most stability. Three varieties displayed 100-kernel weights surpassing the overall environmental average, with A1 and A5 once again showing enhanced stability. Over the two years, most of the four varieties performed better than the overall environmental mean for each trait. However, the number of varieties with good stability was limited, with A1 and A5 showing the highest levels of stability across the various traits.

### 3.6. Correlation Analysis of Agronomic and Physiological Traits in Maize Varieties

**Figure 8:**
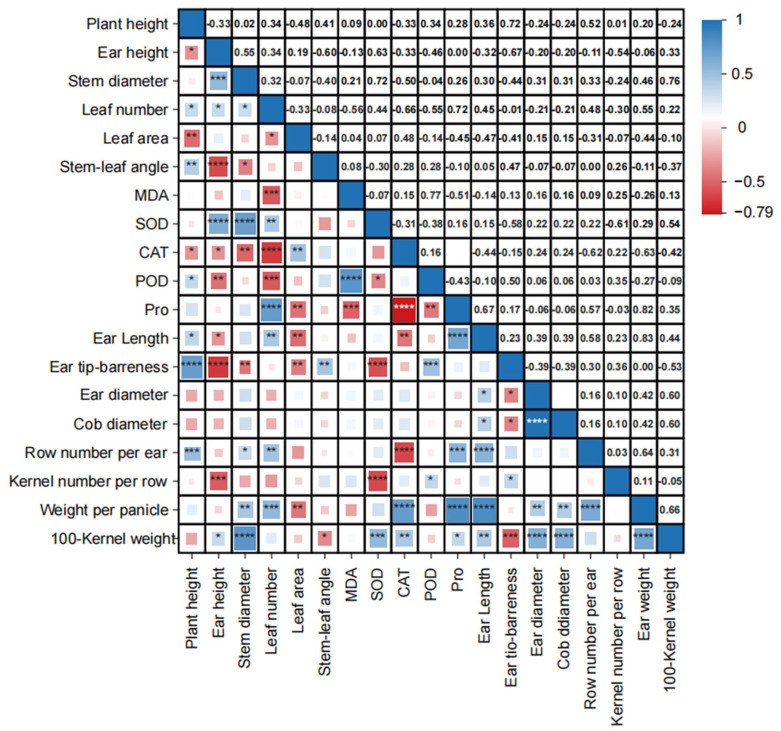
Correlation analysis of the comprehensive traits of the maize varieties. Note: **~**** represents a very significant correlation (*p* < 0.01); * represents a significant correlation (*p* < 0.05).

The correlation analysis of the comprehensive traits in the maize varieties (Figure 8) revealed several statistically significant relationships. Plant height showed a statistically significant negative correlation with ear height and leaf area while exhibiting a statistically significant positive correlation with the leaf number and the stem–leaf angle. Ear height, in turn, had a highly significant positive correlation with plant height and a statistically significant negative correlation with the leaf angle at the ear level. There was a significant positive correlation between the 100-kernel weight and stem diameter, CAT, SOD activity, Pro content, ear diameter, and cob diameter, and an extremely significant negative correlation with ear tip-barrenness. Except for ear tip-barrenness, the yield-related traits of maize showed a significant positive correlation. In terms of antioxidant enzymes, POD and MDA showed a significant positive correlation. In contrast, there was a significant negative correlation between CAT, POD, and Pro.

### 3.7. Principal Component Analysis of Different Maize Composite Traits

#### 3.7.1. Principal Component Extraction and Component Loading Matrix

**Figure 9:**
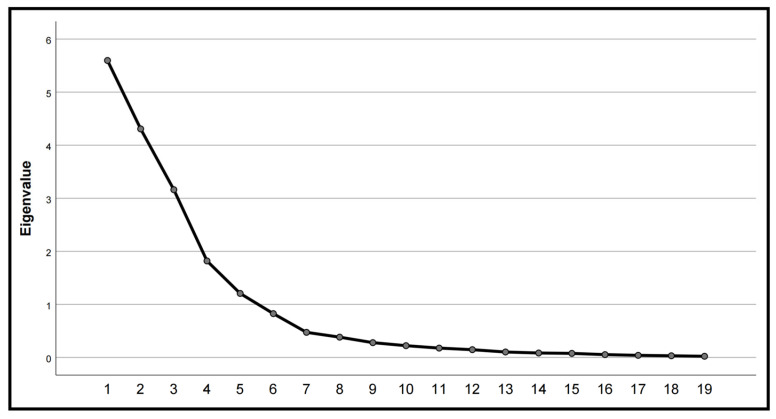
Adaptive evaluation of the comprehensive traits of the different maize varieties by principal component lithotripsy map. Note: The trait value is represented on the vertical axis, and the horizontal axis shows 19 traits. The digits 1~19 represent various plant characteristics, including plant height, ear height, stem diameter, leaf number, leaf area, stem–leaf angle, malondialdehyde (MDA), superoxide dismutase (SOD), catalase (CAT), peroxidase (POD), proline, ear length, ear tip-barrenness, ear diameter, cob diameter, row number per ear, kernel number per row, ear weight, and 100-kernel weight.

As shown in Table 6, the KMO statistic exceeds 0.5, and Bartlett’s test of sphericity is statistically significant (*p* < 0.5), indicating that the correlation between the test variables is strong enough to justify conducting a factor analysis. Figure 9 illustrates that the eigenvalues of the five principal components decrease steeply after the fifth component; these five principal components were selected for further analysis.

As seen in Table 7, the cumulative variance contribution rate of the first five principal components is 84.869%, meaning they account for 84.869% of the information from the original variables. The analysis of the common factor variances shows that the majority of the common variances for each trait exceed 0.5, with most surpassing 0.8 [19,48]. This suggests that the five common factors accurately reflect the majority of the information contained in the original indicators.

As shown in Table 8, the first principal component, known as the “100-kernel weight factor”, has the highest absolute load of 5.596. The second principal component becomes the “ear tip-barrenness factor” with an eigenvalue of 4.307. The third principal component is the “leaf number factor”, which has the highest agreement with the number of leaves. The fourth principal component is the “CAT activity factor”, whose eigenvalue is 3.162. The fifth principal component was the relative load of the “stem–leaf angle factor”, which was 1.206.

#### 3.7.2. Evaluation of Principal Component Scores and Composite Scores

As shown in Table 8, the scores for the principal component factors can be calculated using the following formula based on the matrix of factor score coefficients and their corresponding principal components [48]:

F1 = − 0.02X1 + 0.044X2 + 0.116X3 + 0.065X4 − 0.071X5 + 0.031X6 − 0.126X7 + 0.001X8 − 0.101X9 − 0.102X10 + 0.113X11 + 0.106X12 − 0.079X13 + 0.146X14 + 0.105X15 + 0.08X16 − 0.017X17 + 0.147X18 + 0.159X19;

F2 = 0.158X1 − 0.176X2 − 0.045X3 + 0.025X4 − 0.13X6 − 0.012X7 − 0.147X8 + 0.066X9 + 0.095X10 − 0.036X11 + 0.164X12 + 0.193X13 + 0.104X14 + 0.012X15 + 0.131X16 + 0.121X17 + 0.11X18 − 0.036X19;

F3 = 0.167X1 + 0.15X2 + 0.08X3 + 0.227X4 − 0.21X5 − 0.138X6 + 0.005X7 + 0.205X8 + 0.018X9 − 0.008X10 − 0.113X11 + 0.007X12 + 0.069X130.043X14 − 0.194X15 − 0.016X16 − 0.173X17 + 0.018X18 − 0.047X19;

F4 = 0.06X1 − 0.083X2 − 0.089X3 + 0.096X4 + 0.106X5 − 0.123X6 + 0.282X7 + 0.001X8 − 0.101X9 − 0.102X10 + 0.113X11 + 0.077X12 − 0.074X13 + 0.046X14 + 0.099X15 + 0.08X16 − 0.174X1 − 0.001X18 − 0.146X19;

F5 = 0.065X1 + 0.03X2 + 0.256X3 − 0.233X4 − 0.336X5 + 0.36X6 + 0.318X7 − 0.21X8 − 0.124X9 + 0.304X10 − 0.246X11 + 0.009X12 − 0.026X13 − 0.165X14 + 0.154X15 − 0.079X16 − 0.006X17 − 0.006X18 + 0.121X19;

In this formula, the variables X1~X19 represent various plant characteristics, including plant height, ear height, stem diameter, leaf number, leaf area, stem–leaf angle, malondialdehyde (MDA), superoxide dismutase (SOD), catalase (CAT), peroxidase (POD), proline, ear length, ear tip-barrenness, ear diameter, cob diameter, row number per ear, kernel number per row, ear weight, and 100-kernel weight. The variables F1~F5 represent the scores of each principal component.

A comprehensive score function for evaluating the adaptability of the comprehensive traits in the different maize varieties was constructed by combining the principal component factor score formula, with the variance contribution rate of each principal component as the weight. The formula is as follows:

F = 0.29455F1 + 0.52123F2 + 0.68766F3 + 0.7834F4 + 0.84689F5

Here, F represents the composite score for evaluating the comprehensive trait adaptation of the maize varieties.

The comprehensive evaluation score for the different maize varieties can be calculated based on their growth and adaptability across multiple traits. The ranking of the comprehensive scores is determined by the *F* value, which reflects the overall growth and adaptability of each maize variety. The results are presented in Table 9.

In terms of the comprehensive ranking of the seven maize varieties, A3 achieved the highest evaluation score, followed by A5, A6, A7, A1, A2, and A4. The composite scores for A3 and A5 were 245.84 and 244.86, respectively, indicating a strong similarity and superior performance compared to the other varieties. In contrast, A4 had the lowest composite score of 215.35, indicating a lower level of performance and adaptability under the prevailing environmental conditions in the southern border region.

#### 3.7.3. Principal Component-Based Two-Dimensional Ranking Analysis

**Figure 10:**
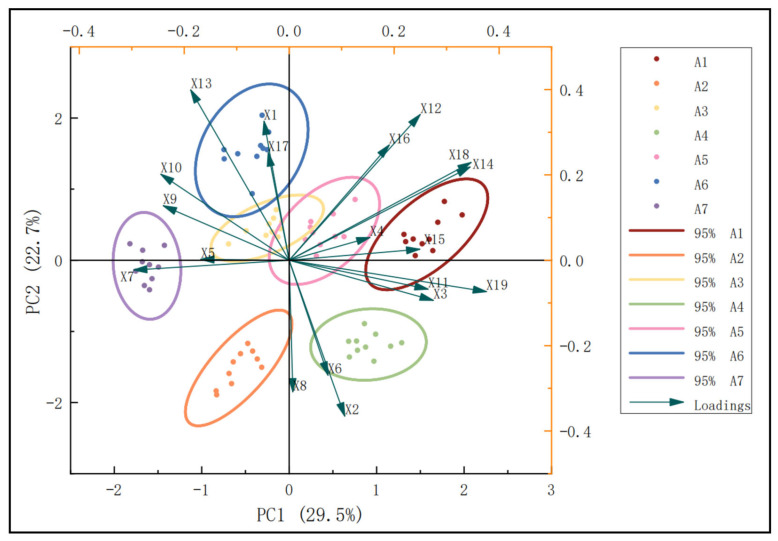
Two-dimensional principal component ranking map for comprehensive trait adaptability evaluation of different maize varieties. Note: X1, X2, X3, X4, X5, X6, X7, X8, X9, X10, X11, X12, X13, X14, X15, X16, X17, X18, and X19 are plant height, ear height, stem diameter, leaf number, leaf area, stem–leaf angle, MDA, SOD, CAT, POD, Pro, ear length, ear tip-barrenness, ear diameter, cob diameter, row number per ear, kernel number per row, ear weight, and 100-kernel weight.

Principal component analysis was used to evaluate the adaptability of the different maize varieties across comprehensive traits (Figure 10). A two-dimensional ordination plot was constructed, with principal component 1 (primarily represented by 100-kernel weight) as the horizontal axis, and principal component 2 (mainly represented by ear tip-barrenness) as the vertical axis. In this context, higher values of 100-kernel weight and ear tip-bareness are considered more important depending on the quadrant where these traits are located. Varieties A5 and A1 are positioned closest to the 100-kernel weight loading line, indicating the strongest correlation with this trait. The degree of correlation is determined by how close a variety is to the 100-kernel weight and ear tip-barrenness loading line, with A3, A6, A4, A7, and A2 also showing proximity to this line.

Meanwhile, the horizontal axis in Figure 10, which represents principal component 1, shows that stem diameter, Pro, ear diameter, cob diameter, ear tip-bareness, row number per row, ear weight, and 100-kernel weight have high loading values (>1). Similarly, the vertical axis, representing principal component 2, illustrates that plant height, POD, ear length, cob diameter, ear weight, and 100-kernel weight also exhibit high loading values (>1). In conclusion, the varieties that displayed superior growth adaptability to the environmental conditions of the southern border region were A1, A3, A5, and A6. On the other hand, A2, A4, and A7 showed poorer adaptation to these environmental conditions.

### 3.8. Cluster Analysis

**Figure 11:**
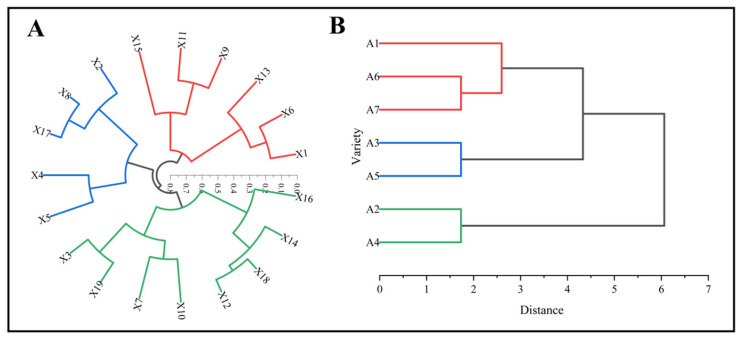
R-type clustering and Q-type clustering for comprehensive trait adaptability evaluation of different maize varieties. Note: In the figure, (**A**,**B**) represent R-type clustering and Q-type clustering. X1, X2, X3, X4, X5, X6, X7, X8, X9, X10, X11, X12, X13, X14, X15, X16, X17, X18, and X19 are plant height, ear height, stem diameter, leaf number, leaf area, stem–leaf angle, MDA, SOD, CAT, POD, Pro, ear length, ear tip-barrenness, ear diameter, cob diameter, row number per ear, kernel number per row, ear weight, and 100-kernel weight.

In this experiment, systematic cluster analysis was applied based on the results of the principal component analysis. R-type clustering was performed on the comprehensive traits of the different maize varieties (Figure 11A), while Q-type clustering was used for the seven maize test varieties (Figure 11B). The intergroup linkage method was employed, with the clustering interval defined by squared Euclidean distance, as shown in Figure 11.

As depicted in Figure 11A, the 19 comprehensive traits of the maize varieties were grouped into three categories. The first category included plant height, ear leaf angle, CAT, Pro, ear tip-barrenness, and cob diameter. The second category consisted of ear height, leaf number, leaf area, SOD, and kernel number per row. The third category comprised stem diameter, MDA, POD, ear length, ear diameter, row number per ear, ear weight, and 100-kernel weight. Based on the principal component loadings, 100-kernel weight, ear tip-barrenness, CAT, stem–leaf angle, and leaf number were selected as the key indices for comprehensively evaluating the adaptive growth of different maize varieties. In Figure 11B, when the clustering number is set to three, the maize varieties are divided into three categories. The first category includes A1, A7, and A6. The second category consists of A3 and A5. The third category contains A2 and A4.

## 4. Discussion

### 4.1. A Comprehensive Comparison of Traits Allows for a Comprehensive Analysis of the Adaptability of Maize Varieties from Different Sources

Xinjiang is a major maize-producing region in China. However, the widespread saline land and limited agricultural water in this area have greatly hindered the expansion of corn cultivation and yield improvement, especially in the southern border region [49]. Despite these challenges, maize is a salt-tolerant crop, with some varieties able to withstand salt concentrations below 0.24% without adverse effects on growth. Certain studies hypothesize that the lethal threshold for salt content is approximately 0.49% [50]. Maize also demonstrates strong alkali tolerance, making it well suited for low-fertility soils, and exhibits significant adaptability across diverse soil conditions [51]. Cultivation variability within the same region, influenced by geographic origin, germplasm development pathways, and varietal traits, suggests considerable genetic diversity in maize germplasm, particularly regarding phenotypic traits [52]. Data indicate that Deng Hai 3672, cultivated in the Aral region of South Xinjiang, has a shorter fertility period and more stable yield-related traits than Jin Ai 588. Moreover, the plant traits of Deng Hai 3672 show enhanced stability. Through comprehensive evaluation, Deng Hai 3672 was found to exhibit stable growth characteristics and high CAT activity in this region, helping to prevent excessive ROS accumulation and reduce oxidative damage under salt and alkali stress [53]. This suggests that Deng Hai 3672 may be particularly effective when introduced into areas facing saline stress. Compared with other corn varieties, especially the Xin Yu 66 varieties, the MDA content of the Jin Ai 588 varieties was significantly reduced, but the antioxidant enzyme content was not high, including SOD, CAT, and POD. Jin Ai 588 showed strong proline accumulation ability. Nevertheless, it is important to consider the impact of additional climatic factors. The city of Alar in Southern Xinjiang, located on the desert’s edge, experiences very low annual precipitation, making maize susceptible to drought stress. Wu et al. [54] study found a correlation between leaf area and drought tolerance in maize varieties, indicating that maintaining sufficient green leaf area is essential for achieving high yields [55]. Tóth et al. [56] demonstrated that traits such as ears, stems, and leaves can be quantified and analyzed to optimize maize plant morphology and improve light capture, thereby improving yield potential. During the 2021–2022 period, the Xin Yu 66 and Deng Hai 3672 varieties showed greater stability in plant traits. The Xin Yu 66 variety, in particular, displayed increased plant height, stem diameter, and leaf number across both years, along with superior growth characteristics, including a stem–leaf angle of less than 30° and a more compact plant structure. These factors likely contributed to more efficient photosynthesis and organic matter accumulation, leading to higher yields [57].

Germplasm resources form the fundamental basis of breeding. Analyzing the genetic diversity of various traits within germplasm resources can accelerate the breeding process. In this study, the coefficient of variation for ear height among the plant morphological traits was 10.2%, aligning closely with Guo et al.’s [58] findings but differing from those of Meng et al. [59]. This discrepancy may stem from variations in climate, variety types, and cultivation conditions between the two regions. The coefficients of variation for bald tip-barrenness, ear weight, and 100-kernel weight were all above 10%, suggesting these traits exhibit significant genetic variability and high heritability.

### 4.2. A Comprehensive Evaluation of the Stability of Maize Variety Will Be Conducted Using Multiple Analytical Methods

The correlation analysis revealed a significant positive relationship between stem diameter and 100-kernel weight, as well as between ear weight and 100-kernel weight. Additionally, a highly significant positive correlation was observed between 100-kernel weight and SOD activity, consistent with the findings of Wang et al. [60]. This correlation may be due to the role of antioxidant enzymes in scavenging intracellular long-chain free radicals and H₂O₂, which have been associated with delayed senescence in crop plants [61]. MDA content and proline content are closely linked to plant senescence and stress tolerance, often used as indicators of the degree of leaf senescence in plants. Previous studies have shown that plants can reduce the effects of cellular aging and membrane disruption by increasing proline content and soluble protein levels in response to stressful conditions [62]. MDA, a byproduct of membrane lipid peroxidation, serves as an indicator of damage from environmental stress: higher levels suggest increased damage, while lower levels suggest reduced stress impact [63]. The study found a significant positive correlation between proline content and both ear weight and 100-kernel weight, whereas MDA levels were negatively correlated with ear weight and 100-kernel weight. Jin Ai 588 demonstrated the best performance in these traits, with the lowest MDA content of 40.35 nmol/mg and the highest proline content of 26.33 ng/g. This suggests that the adaptability and overall performance of maize varieties are not solely determined by agronomic traits or physiological traits. Instead, they result from the interplay and coordination of multiple traits.

The stability of crop germplasm resources is contingent upon the consistency of various agronomic traits, which are shaped by genotype–environment interactions. Among the analytical methods available, GGE biplots are especially valuable for identifying genotypes that perform optimally at specific locations or exhibit stability across diverse environments [64,65]. Moreover, GGE biplots aid in pinpointing the most representative location for a particular genotype. Their effectiveness in visualizing relationships among varieties, traits, and populations has been highlighted by Kendel et al. [66]. In this study, the first (PC1) and second (PC2) principal components were utilized to generate a GGE biplot for the seven maize hybrids, providing insights into the stability of agronomic and physiological traits from 2021 to 2022. The analysis showed that Xin Yu 66 and Deng Hai 3672 showed good stability in plant type and physiological traits, and Jin Ai 588 and Deng Hai 3672 showed good stability in yield-related traits. The results indicated that these three varieties had good growth adaptability under the environmental conditions in the southern border area, and were suitable for widespread planting in this area to support the efficient utilization of high-quality maize germplasm resources.

Principal component analysis (PCA) is a widely recognized and objective statistical method that reduces data dimensionality by transforming numerous indicators into a smaller set of independent composite indicators. It has been extensively applied for the comprehensive evaluations of various crops across multiple dimensions [67]. Crop traits often show complex interrelationships and constraints, making it challenging to achieve a complete and precise evaluation using individual indicators alone. PCA addresses this complexity by condensing multiple variables into key factors, effectively handling large datasets. In light of these considerations, employing multivariate statistical techniques is essential for evaluating and identifying key indicators [68]. The analysis of principal components and composite scores indicated that the cumulative contribution rate of the first five principal components reached 84.689% through the analysis of the 19 traits in evaluating the performance and suitability of the seven maize varieties. These components collectively represented 86.689% of the full range of traits, simplifying the comparison of traits among varieties and improving the efficiency of screening and evaluation. The comprehensive evaluation results identified five principal components reflecting, in turn, the 100-kernel weight, bald tip-barrenness, CAT activity, leaf number, and stem–leaf angle in maize. Among the varieties evaluated, Xin Yu 66 achieved the highest comprehensive score, making it the most suitable for cultivation in the Aral region of Southern Xinjiang. The score difference between Xin Yu 66 and Deng Hai 3672 was not statistically significant, suggesting that both varieties share similar traits contributing to their suitability for this region. The combined scores for Jin Ai 588, Gan Xin 2818, Xin Yu 81, and Zheng Dan 958 were relatively low, indicating that these varieties have the potential for broader application in this area. In contrast, Xin Yu 24 exhibited the lowest overall performance in terms of growth suitability, suggesting limited utility for cultivation in the Alar region of South Xinjiang. Furthermore, it has been shown that the climate factors experienced when sowing at the optimal time significantly influence dry matter accumulation dynamics, photosynthetic efficiency, and yield trait variation in maize (*p* < 0.05) [69,70]. Thus, adjusting the sowing period according to each variety’s adaptive performance may effectively increase maize yield and quality.

After applying a clustering technique to analyze the 19 traits of the seven maize germplasm materials, it was found that these maize varieties could be grouped into three distinct clusters. By combining the R-type clustering results with the principal component loading analysis, five key indices were selected for a comprehensive evaluation of maize suitability for planting in the Alar area of South Xinjiang: 100-kernel weight, bald tip-barrenness, CAT activity, leaf number, and stem–leaf angle. These indices can be broadly categorized into plant morphology traits, physiological traits, and yield-related traits. The Q-clustering results indicate that the first category of maize varieties, represented by CAT (including Xin Yu 66 and Deng Hai 3672), is characterized by a compact growth pattern with a moderate leaf number, as demonstrated by the leaf number and the stem–leaf angle. These traits suggest that Xin Yu 66 and Deng Hai 3672 are well suited for cultivation in this region. The second category, consisting of the varieties Jin Ai 588, Gan Xin 2818, and Xin Yu 81, is distinguished by a bald tip length greater than 10 mm. Despite this trait, they also display longer ear lengths and a higher kernel number per row, which makes them suitable candidates as one parent in specific germplasm breeding programs. Finally, the third category is defined by 100-kernel weight and includes the varieties Zheng Dan 958 and Xin Yu 24. While these varieties do not show outstanding yield-related traits, they have distinct ear heights and larger leaf areas compared to the other varieties, making them potentially useful as parental options in genetic improvement programs.

### 4.3. Variety Selection and Application Broaden the Selection of Germplasm Resources

Basic research on maize in Southern Xinjiang is still in its nascent stages, with limited advancements at both macro and micro levels. Varieties in the region often show instability in agronomic and genetic traits, and there is a pronounced lack of high-quality, multi-resistant, and broadly adaptable varieties. Additionally, the genetic base of available germplasm resources is narrow, with limited diversity, which restricts the genetic foundation essential for both research and breeding [71]. The effective evaluation of germplasm resources is critical for advancing germplasm innovation and developing new varieties, as comprehensive assessments form a key step in improving the existing varieties [72]. In this study, an analysis of 19 traits across seven maize varieties over a two-year period revealed year-to-year variations, mainly due to environmental factors. Specifically, precipitation levels in 2022 were significantly lower than in 2021, contributing to these observed differences. From the analysis, two varieties, Xin Yu 66 and Deng Hai 3672, were identified as having superior physiological adaptability and resistance, making them particularly suitable for propagation and cultivation in Southern Xinjiang. Their adoption could increase the effective use of high-quality maize germplasm resources in the region. These findings also provide valuable theoretical insights for the introduction of maize varieties to Southern Xinjiang and support the broader application of germplasm resources.

## 5. Conclusions

Over the study period, the seven maize varieties displayed significant diversity and variation in each trait. The correlation analysis revealed complex interdependencies and constraints among these traits. The stability of the 19 traits in two years was analyzed by GGE double marking. The principal component analysis identified five key principal components from the 19 traits. The cluster analysis then categorized the maize varieties into three groups in order to provide the dominant germplasm resources of different groups as breeding materials in the breeding process of maize germplasm in Alar and similar climate areas in Southern Xinjiang so as to effectively improve the utilization rate of germplasm resources.

## Figures and Tables

**Table 1:** Experimental materials.

Number	Variety	Source	Pedigree	Growth Period (Days)
A1	Jin Ai 588	Neimenggu	823, 155 × 823, 181	125
A2	Zheng Dan 958	Henan	Zheng58 × Chang7-2	130
A3	Xin Yu 66	Xinjiang	4482 × DM17	125
A4	Xin Yu 24	Xinjiang	TH78 × TH189	123
A5	Deng Hai 3672	Shandong	DH13 × DH101	120
A6	Gan Xin 2818	Gansu	Wu9086 × 6073	139
A7	Xin Yu 81	Xinjiang	W17 × HA1M	133

**Table 2:** Combined analysis of maize varieties and years and their interaction effects.

Traits	Year	Variety	Year × Variety
DF	1	6	6
Plant height	18.41 **	35.90 **	49.10 **
Ear height	58.22 **	73.93 **	7.49 **
Stem diameter	6505.48 **	16.04 **	11.61 **
Leaf number	0.50	28.71 **	35.11 **
Leaf area	2324.67 **	62.64 **	48.93 **
Stem–leaf angle	16.80 **	15.26 **	14.69 **
MDA	793.41 **	50.41 **	5.79 **
SOD	743.78 **	62.88 **	6.87 **
CAT	1260.11 **	51.00 **	9.29 **
POD	5662.67 **	242.86 **	36.61 **
Pro	48.27 **	25.1 **	3.77 **
Ear length	401.58 **	17.95 **	16.14 **
Ear tip-barrenness	1009.11 **	86.05 **	84.12 **
Ear diameter	185.35 **	33.86 **	5.12 **
Cob diameter	42.05 **	16.09 **	10.64 **
Row number per ear	0.41 **	7.91 **	2.88 *
Kernel number per row	94.86 **	5.24 **	1.57
Ear weight	1891.72 **	59.71 **	34.06 **
100-Kernel weight	4952.22 **	137.68 **	28.93 **

Note: * respent significant at the 0.05 level; ** respent significant at the 0.01 level.

**Table 3:** Diversity analysis of 6 morphological traits in 7 maize varieties in 2 years.

Traits	The Average of the Varieties							Max	Min	Mean	SD	CV (%)
	A1	A2	A3	A4	A5	A6	A7					
Plant height (cm)	261.51 bc	250.09 e	276.45 a	252.40 d	256.03 c	268.93 b	259.74 c	276.45	250.09	260.74	9.31	3.6
Ear height (cm)	108.74 b	121.12 a	109.41 b	121.04 a	94.75 c	97.08 c	98.91 c	121.12	94.75	107.29	10.94	10.2
Stem diameter (mm)	19.44 ab	18.73 b	19.53 ab	20.38 a	18.94 b	18.61 b	17.66 b	20.38	17.66	19.04	0.86	4.5
Leaf number (piece)	15.23 a	14.30 ab	14.77 ab	13.53 bc	12.98 c	14.05 c	13.03 c	15.23	12.98	13.99	0.85	6.1
Stem–leaf angle (°)	31.39 c	31.60 bc	29.84 c	30.75 c	33.74 b	31.02 c	35.26 a	35.26	29.84	31.94	1.89	5.9
Leaf area (cm^2^)	488.45 c	545.25 a	469.59 d	543.66 a	535.95 a	505.09 b	486.98 cd	545.25	510.71	510.71	30.81	6.0

Note: (a–d) markers of significance of differences at 0.05 level.

**Table 4:** Diversity analysis of 5 resistance physiological traits in 7 maize varieties in 2 years.

Traits	The Average of the Varieties							Max	Min	Mean	SD	CV (%)
	A1	A2	A3	A4	A5	A6	A7					
MDA (nmol/mg)	40.35 e	43.2 d	49.13 b	52.13 a	50.24 ab	49.61 b	44.78 c	52.13	52.13	47.06	4.32	9.2
SOD (µ/g)	41.3 b	40.3 bc	44.09 a	42.45 ab	40.3 c	34.83 d	35.29 d	44.09	44.09	39.79	3.49	8.8
CAT (µ/µg)	78.48 c	83.66 b	80.1 bc	80.61 bc	88.97 a	76.68 c	90.57 a	90.57	90.57	82.72	5.28	6.4
POD (µ/g)	240.09 b	226.01 c	265.83 a	269.12 a	259.89 a	272.3 a	268.75 a	272.30	272.30	257.43	17.56	6.8
Pro (ng/g)	26.33 a	22.31 b	22.95 b	21.6 b	21.93 b	25.3 a	20.02 c	26.33	26.33	22.92	2.19	9.6

Note: (a–e) markers of significance of differences at 0.05 level.

**Table 5:** Diversity analysis of 8 yield-related traits in 7 maize varieties in 2 years.

Traits	The Average of the Varieties							Max	Min	Mean	SD	CV (%)
	A1	A2	A3	A4	A5	A6	A7					
Ear length (mm)	20.7 a	18.35 b	19.83 ab	18.97 b	20.14 ab	20.25 a	18.41 b	20.70	18.35	19.52	0.94	4.8
Ear tip-barrenness (mm)	11.85 c	9.95 b	14.5 cd	9.19 d	11.54 c	17.44 a	14.23 b	17.44	9.19	12.67	2.89	22.8
Ear diameter (mm)	54.91 a	41.42 c	47.03 bc	48.19 b	50.21 ab	47.48 b	43.55 bc	54.91	41.42	47.54	4.39	9.2
Cob diameter (mm)	28.31 b	25.2 c	26.18 bc	28.77 ab	30.8 a	25.78 c	26.5 bc	30.80	25.20	27.36	2.00	3.3
Row number per ear	15.8 ab	14.7 b	16 a	15.1 ab	15.3 ab	16.3 a	13.9 c	16.30	13.90	15.30	0.83	5.4
Kernel number per row	39.05 b	37.15 cd	36.85 d	38.60 cd	41.25 a	42.15 a	40.55 ab	42.15	36.85	39.37	2.03	5.2
Ear weight/g	284.57 a	192.82 d	221.36 cd	223.41 cd	240.2 c	248.57 b	163.9 e	284.57	163.90	224.98	38.99	17.3
100-Kernel weight/g	36.85 b	30.89 d	31.61 cd	40 a	35.43 b	32.24 c	27.76 e	40.00	27.76	33.54	4.13	12.3

Note: (a–e) markers of significance of differences at 0.05 level.

**Table 6:** KMO and Barlett sphericity test.

Inspection	Index	Value
KMO	statistics	0.759
Bartlett’s test of sphericity	approx. chi-square	4853.613
	df	171
	sig.	0.000

**Table 7:** Total variance interpretation for comprehensive trait evaluation of different maize varieties.

Principal Component	Initial Eigenvalues			Extraction Sums of Squared Loadings		
	Total	% of Variance	Cumulative %	Total	% of Variance	Cumulative %
P1	5.596	29.455	29.455	5.596	29.455	29.455
P2	4.307	22.668	52.123	4.307	22.668	52.123
P3	3.162	16.643	68.766	3.162	16.643	68.766
P4	1.819	9.574	78.34	1.819	9.574	78.34
P5	1.206	6.349	84.689	1.206	6.349	84.689

Note: P1, P2, P3, P4, and P5 represented the 100-kernel weight, ear tip-barrenness, leaf number, CAT, and stem–leaf angle.

**Table 8:** Adaptive evaluation principal component matrix, rotated principal component matrix, and common factor variance for comprehensive traits of different maize varieties.

Index	Principal Component Matrix ^a^					Principal Component Matrix After Rotation ^b^					Common Factor Variance	
	1	2	3	4	5	1	2	3	4	5	Initial	Extraction
Plant height	−0.113	0.681	0.528	0.109	0.078	−0.02	0.158	0.167	0.06	0.065	1	0.774
Ear height	0.247	−0.756	0.474	−0.152	0.036	0.044	−0.176	0.15	−0.083	0.03	1	0.882
Stem diameter	0.649	−0.193	0.254	−0.161	0.309	0.116	−0.045	0.08	−0.089	0.256	1	0.645
Leaf number	0.366	0.109	0.718	0.175	−0.281	0.065	0.025	0.227	0.096	−0.233	1	0.77
Leaf area	−0.399	−0.002	−0.663	0.193	−0.405	−0.071	0	−0.21	0.106	−0.336	1	0.801
Stem leaf angle	0.173	−0.56	−0.435	−0.223	0.435	0.031	−0.13	−0.138	−0.123	0.36	1	0.772
MDA	−0.703	−0.051	0.015	0.513	0.384	−0.126	−0.012	0.005	0.282	0.318	1	0.906
SOD	0.007	−0.634	0.649	0.23	−0.254	0.001	−0.147	0.205	0.126	−0.21	1	0.94
CAT	−0.565	0.282	0.057	−0.721	−0.15	−0.101	0.066	0.018	−0.396	−0.124	1	0.945
POD	−0.574	0.407	−0.026	0.501	0.367	−0.102	0.095	−0.008	0.275	0.304	1	0.881
Pro	0.63	−0.156	−0.357	0.552	−0.297	0.113	−0.036	−0.113	0.304	−0.246	1	0.942
Ear length	0.595	0.706	0.023	0.141	0.011	0.106	0.164	0.007	0.077	0.009	1	0.873
Ear tip-barrenness	−0.44	0.832	0.217	−0.134	−0.031	−0.079	0.193	0.069	−0.074	−0.026	1	0.953
Ear diameter	0.817	0.45	−0.135	0.084	−0.199	0.146	0.104	−0.043	0.046	−0.165	1	0.935
Cob diameter	0.59	0.05	−0.613	0.179	0.186	0.105	0.012	−0.194	0.099	0.154	1	0.793
Row number per ear	0.45	0.564	0.362	−0.029	0.329	0.08	0.131	0.114	−0.016	0.273	1	0.761
Kernel number per row	−0.093	0.521	−0.547	−0.317	−0.095	−0.017	0.121	−0.173	−0.174	−0.079	1	0.688
Ear weight	0.822	0.473	0.057	−0.001	−0.007	0.147	0.11	0.018	−0.001	−0.006	1	0.902
100-Kernel weight	0.889	−0.156	−0.148	−0.265	0.146	0.159	−0.036	−0.047	−0.146	0.121	1	0.928

Note: The extraction method is principal component analysis; the rotation method is Caesar’s normalized maximum variance method. ^a^ indicates that three principal components are extracted; ^b^ indicates that the rotation has converged after 5 iterations.

**Table 9:** The principal component score coefficient matrix, composite factor score (F), and ranking of adaptive traits of different maize varieties.

Index	Component Score Coefficient Matrix Component					Variety	F1	F2	F3	F4	F5	F	Ranking
	1	2	3	4	5								
X_1_	−0.02	0.158	0.167	0.06	0.065	A1	41.77	27.18	−12.27	2.52	247.08	229.25	5
X_2_	0.044	−0.176	0.15	−0.083	0.03	A2	1.21	5.53	−19.79	2.39	279.24	227.99	6
X_3_	0.116	−0.045	0.08	−0.089	0.256	A3	24.06	27.13	−5.77	14.99	256.03	245.84	1
X_4_	0.065	0.025	0.227	0.096	−0.233	A4	34.90	7.09	−20.52	−13.14	266.59	215.35	7
X_5_	−0.071	0	−0.21	0.106	−0.336	A5	30.69	21.14	−25.81	8.52	278.52	244.86	2
X_6_	0.031	−0.13	−0.138	−0.123	0.36	A6	28.17	29.67	−15.56	2.59	265.06	239.56	3
X_7_	−0.126	−0.012	0.005	0.282	0.318	A7	14.2	17.59	−16.33	6.43	265.06	231.65	4
X_8_	0.001	−0.147	0.205	0.126	−0.21								
X_9_	−0.101	0.066	0.018	−0.396	−0.124								
X_10_	−0.102	0.095	−0.008	0.275	0.304								
X_11_	0.113	−0.036	−0.113	0.304	−0.246								
X_12_	0.106	0.164	0.007	0.077	0.009								
X_13_	−0.079	0.193	0.069	−0.074	−0.026								
X_14_	0.146	0.104	−0.043	0.046	−0.165								
X_15_	0.105	0.012	−0.194	0.099	0.154								
X_16_	0.08	0.131	0.114	−0.016	0.273								
X_17_	−0.017	0.121	−0.173	−0.174	−0.079								
X_18_	0.147	0.11	0.018	−0.001	−0.006								
X_19_	0.159	−0.036	−0.047	−0.146	0.121								

Note: X1, X2, X3, X4, X5, X6, X7, X8, X9, X10, X11, X12, X13, X14, X15, X16, X17, X18, and X19 are plant height, ear height, stem diameter, leaf number, leaf area, stem–leaf angle, MDA, SOD, CAT, POD, Pro, ear length, ear tip-barrenness, ear diameter, cob diameter, row number per ear, kernel number per row, ear weight, and 100-kernel weight.

## Data Availability

Data are contained within the article.
